# The pathogenesis of H7N8 low and highly pathogenic avian influenza viruses from the United States 2016 outbreak in chickens, turkeys and mallards

**DOI:** 10.1371/journal.pone.0177265

**Published:** 2017-05-08

**Authors:** Mary J. Pantin-Jackwood, Christopher B. Stephens, Kateri Bertran, David E. Swayne, Erica Spackman

**Affiliations:** Exotic and Emerging Avian Viral Diseases Unit, Southeast Poultry Research Laboratory, U.S. National Poultry Research Center, U.S. Department of Agriculture, Agricultural Research Service, Athens, Georgia, United States of America; Centers for Disease Control and Prevention, UNITED STATES

## Abstract

In January 2016, a combined outbreak of highly pathogenic (HP) avian influenza virus (AIV) and low pathogenicity (LP) AIV occurred in commercial turkeys in the state of Indiana, United States. Genetically, the viruses were highly similar, belonged to the North American wild bird lineage, and had not been previously detected in poultry. In order to understand the pathobiology of the H7N8 LPAIV and HPAIV, infectivity, transmission and pathogenicity studies were conducted in chickens, turkeys, and mallards. Among the three species the lowest mean infectious dose for both the LP and HP phenotype was for turkeys, and also disease from the LPAIV was only observed with turkeys. Furthermore, although the HPAIV was lethal for both chickens and turkeys, clinical signs caused by the HPAIV isolate differed between the two species; neurological signs were only observed in turkeys. Mallards could be infected with and transmit both viruses to contacts, but neither caused clinical disease. Interestingly, with all three species, the mean infectious dose of the HP isolate was at least ten times lower than that of the LP isolate. This study corroborates the high susceptibility of turkeys to AIV as well as a pathobiology that is different from chickens. Further, this study demonstrates that mallards can be asymptomatically infected with HP and LP AIV from gallinaceous poultry and may not just be involved in transmitting AIV to them.

## Introduction

Wild aquatic birds are the natural reservoirs of avian influenza virus (AIV) and usually carry the low pathogenic (LP) phenotype [1]. Periodically, AIV can transmit from wild birds to domestic birds resulting in subclinical infections or respiratory disease and drops in egg production [2]. After circulating in gallinaceous poultry (e.g. chickens and turkeys), some H5 and H7 LPAIV isolates can mutate to the highly pathogenic (HP) phenotype, which causes severe systemic disease and high mortality in gallinaceous bird species but typically does not cause disease in ducks [3] (the HP phenotype is defined by virulence in chickens [4]).

On January 16, 2016, an H7N8 HPAIV infection was confirmed in a turkey flock on a single farm in the state of Indiana, United States (US), and LPAIV of the same subtype was subsequently detected in nine nearby turkey flocks [5]. Initial gene sequence analysis showed that the HPAIV was closely related to the LPAIVs and was likely derived from them [5], but no similar virus has been previously found in poultry. Sequence analysis of the HA genes also showed that these viruses were closely related to a recent wild duck origin H7N8 LPAIV [5, 6]. Therefore, a LPAIV was likely transmitted from wild aquatic birds to the turkeys and subsequently mutated to HPAIV within the turkey population. Fortunately, the outbreak was immediately controlled and no further premises were affected, and no human infections were identified.

Because AIV pathobiology varies among strains and host species, characterizing the pathobiology of new viruses in relevant bird species is crucial to understanding the epidemiology of AIVs and effectively controlling them [7]. Comparisons of the relative susceptibility and pathogenicity of HPAIV or LPAIV in different avian species have been reported numerous times. However, there are few direct comparisons of an LP-HP isolate pair side-by-side with three of the most economically and ecologically important avian species affected by AIV (i.e. chickens [*Gallus gallus domesticus*], turkeys [*Meleagris gallopova*] and mallards [*Anas platyrhynchos*]) [8]. In this study, we evaluate the infectious dose, pathogenesis, virus shed (amount of virus excreted) and transmission dynamics of LP and HPAIV H7N8 isolates from the 2016 US outbreak in these three avian species.

## Materials and methods

### Viruses

Egg passage 1 stocks of A/turkey/Indiana/16-001571-6/2016 (H7N8) LPAIV (GenBank accession numbers KY684308-KY684315) and A/turkey/Indiana/16-001403-1/2016 (H7N8) HPAIV (GenBank accession numbers KU558903-KU558910 [5]) were provided by the National Veterinary Services Laboratories, USDA-APHIS. Working stocks were prepared (egg passage 2) and titrated in embryonating chickens eggs using standard methods [9]. Stocks were diluted to the target dose with brain-heart infusion (BHI) broth (Becton, Dickinson and Company, Sparks, MD).

### Animals and housing

Day-old commercial broad-breasted white turkeys and mallards were obtained from commercial producers and reared in animal biosafety level (ABSL) 2 enhanced facilities at the US National Poultry Research Center, USDA-ARS (USNPRC). Specific pathogen free (SPF) white-leghorn chickens were obtained from USNPRC in-house flocks. At 2 weeks of age (mallards) or 3 weeks of age, (chickens and turkeys) birds were transferred to ABSL-3 enhanced facilities for challenge. Ten chickens, turkeys and mallards were bled immediately prior to challenge to confirm the absence of AIV antibody with a commercial ELISA (AI MultiS-Screen, IDEXX, Westbrook, ME). Each experimental group was housed in self-contained isolation units ventilated under negative pressure with inlet and exhaust HEPA-filtered air. This study and associated procedures were reviewed and approved by the USNPRC Institutional Animal Care and Use committee. The study was conducted in accordance with the recommendations of the Federation of Animal Science Societies Guide for the Care and Use of Agricultural Animals in Research and Teaching, 3^rd^ Edition. Humane endpoints were used and euthanasia was the primary method to minimize suffering and distress. Euthanasia is applied to birds that are moribund. Birds are considered moribund if they meet one or more of the following criteria: 1) impaired ambulation which prevents animals from reaching food or water; 2) lack of physical or mental alertness; 3) difficult, labored breathing; and 4) inability to remain upright. Turkeys and chickens were euthanized by cervical dislocation. Mallards were euthanized by intravenous administration of sodium-pentobarbital 100mg/Kg body weight. Animals were monitored twice daily when disease was apparent and once daily when the birds were healthy. No unexpected deaths or clinical signs were observed.

### Infectivity, transmission and pathogenesis studies in chickens, turkeys and mallards

Chicken and turkey studies were conducted identically and simultaneously (i.e. the same inocula preparations were used for both species). Mallard studies were conducted with inoculum prepared at a different time and with a few minor modifications because of the expected differences in AIV pathobiology in the mallards, as noted below. Birds were divided into groups as shown in Table 1 and individually tagged for identification. The inoculum was prepared by diluting a working virus stock to approximately 10^6^ 50% egg infectious doses (EID_50_) (high dose), 10^4^ EID_50_ (medium dose), and 10^2^ EID_50_ (low dose) in 0.1ml and was administered by the intra-choanal route. The actual titers of the inocula were confirmed by titration in ECE and were within 0.5 log_10_ of the target titer for all groups.”Sham exposed birds were inoculated with 0.1 ml of sterile allantoic fluid diluted 1:300 in BHI media. To evaluate transmission by contact exposure, non-inoculated hatch-mates (contacts) were added to each dose group 24 hours (hr) post inoculation (PI).

**Table 1:** Mortality, mean death times, total birds infected and 50% bird infectious doses for chickens, turkeys and mallards inoculated with H7N8 LPAIV or H7N8 HPAIV. Total infected contact exposed birds are also shown.

		**Chickens**				**Turkeys**				**Mallards**			
		Inoculated			Contact Exposed	Inoculated			Contact Exposed	Inoculated			Contact Exposed
Isolate	Log_10_ Dose	Mortality (MDT^b^)	Infected/ Total^c^	BID_50_^d^	Infected/ total	Mortality (MDT)	Infected/ total	BID_50_	Infected/ total	Mortality (MDT)	Infected/total	BID_50_	Infected/ total
LPAIV A/turkey/IN/16-001571-6/2016	2	0/5^c^	0/5	10^4.0^ EID_50_^e^	0/3	0/5	0/5	10^3^ EID_50_	0/3	0/5	0/5	10^3.6^ EID_50_	0/3
	4	0/5	3/5		0/3	0/5	5/5		3/3	0/5	3/5		0/3
	6^a^	0/17	17/17		0/3	0/17	17/17		3/3	0/8	8/8		3/3
HPAIV A/turkey/IN/16-001403-1/2016	2	0/5	2/5	10^3.2^ EID_50_	0/3	5/5 (156.8hr)	5/5	<10^2^ EID_50_	3/3	0/5	2/5	10^2.5^ EID_50_	1/3
	4	4/5 (60hr)	5/5		0/3	5/5 (81.6hr)	5/5		3/3	0/5	4/5		1/3
	6	16/17 (66hr)	17/17		0/3	17/17 (67.8hr)	17/17		3/3	0/8	8/8		3/3

a. The high dose groups of chickens and turkeys were housed in a group of 5 inoculates with 3 contact exposure birds and a group of 12. The 10^6^ dose groups of mallards were housed in a group of 8 inoculates with 3 contact exposure birds.

b. MDT = Mean death time in hours if applicable.

c. Birds were considered infected if they shed virus and/or were positive for antibodies two-weeks post-exposure.

d. BID_50_ = 50% bird infectious dose.

e. EID_50_ = 50% egg infectious doses.

Oropharyngeal (OP) and cloacal (CL) swabs were collected from directly inoculated chickens and turkeys in the high dose group at 12hr, 24hr, 36hr, 48hr, 3 days (d), 4d, 7d and 10d PI. Oropharyngeal swabs included the buccal cavity and choanal cleft. Swabs (OP and CL) were collected from directly inoculated chickens and turkeys in the medium and low dose groups at 24hr, 48hr, 3d and 4d PI. Oropharyngeal and CL swabs were collected from contact chickens and turkeys at 24hr, 48hr, 3d and 4d after placement with the inoculated birds. Oropharyngeal and (CL) swabs were collected from mallards at 2, 4, 7, and 10d PI. Swabs were placed in 2ml of BHI broth with penicillin (2000 units/ml; Sigma Aldrich), gentamicin (200 μg/ml; Sigma Aldrich) and amphotericin B (5 μg/ml; Sigma Aldrich). The swab material was stored at -80C until it was processed.

At 48hr PI two inoculated chickens and turkeys in the high dose group were euthanized and necropsied to examine for gross lesions and collect tissues for microscopic evaluation and visualization of viral antigen by influenza specific immunohistochemical (IHC) staining. Two sham exposed hatch-mates were also euthanized and necropsied as control birds. At 3d PI, three inoculated and 2 sham-inoculated mallards were euthanized for necropsy. A full set of tissues was collected from each bird and fixed in 10% neutral buffered formalin solution, paraffin-embedded, sectioned, and stained with hematoxylin-and-eosin. Duplicate sections were stained by IHC methods to visualize the distribution of influenza virus antigen in individual tissues [10]. Lung, spleen, heart, muscle and brain were also collected and frozen at -80°C for subsequent virus detection. Birds that died or were euthanized for humane reasons were also necropsied to evaluate gross lesions. Birds that were euthanized were counted as dead at the next observation time for mean death time (MDT) calculations.

Body temperatures and body weights were taken from inoculated mallards in the high dose groups and from sham-inoculated mallards at 2 and 4d PI.

Sera were collected from all surviving birds at 14d PI to evaluate infection status by antibody levels using hemagglutination inhibition (HI) assay. Hemagglutination assay was performed using standard methods and homologous antigen [11].

The virus infectious dose was calculated by the Reed-Muench method [12], using the criteria that birds were considered infected if they shed detectable levels of virus at any time and/or were positive for antibody at 14d PI.

### Quantitative real-time RT-PCR

Total RNA from swabs and tissues was tested by quantitative real-time RT-PCR (qRRT-PCR) targeting the influenza M gene as previously described [13]. Virus titers in tissue samples were determined by weighing, homogenizing tissues, and diluting in BHI broth to a 10% (wt/vol) concentration. Total RNA was extracted from tissues using Trizol LS reagent (Invitrogen, Carlsbad, CA) and the Qiagen RNeasy Mini Kit (Qiagen Corp, Valencia, CA) was used to recover RNA from the aqueous phase instead of precipitation. Equal amounts of RNA extracted from the tissue samples were used in the qRRT-PCR assay (50 ng/μl). The standard curves for swabs and tissues were run in triplicate using RNA from the same virus stock used to prepare the inoculum. Virus quantity was reported as equivalents to infectious titer. The calculated qRRT-PCR lower detection limit for the viruses in OP and CL swabs was 10^1.6^ EID_50_/ml (chicken and turkey samples), and 10^1.9^ EID_50_/ml (mallard samples). The threshold of detection in tissue samples was 10^1.5^ EID_50_/g.

### Statistics

Shed titers by time point for the same swab type (OP or CL) were analyzed between the HPAIV and LPAIV and among species with the Mann-Whitney Rank Sum test (SigmaPlot 13.0, Systat Software, San Jose, CA). If virus was not detected in a sample, it was given the value of 0.1 log_10_ below the qRRT-PCR test limit of detection. Proportions of birds shedding were tested with Fisher’s exact test (SigmaPlot 13.0). A p value of ≤0.05 was considered significant.

## Results

### Infectivity, transmission and pathogenicity of the H7N8 LPAIV in chickens

**Fig 1:**
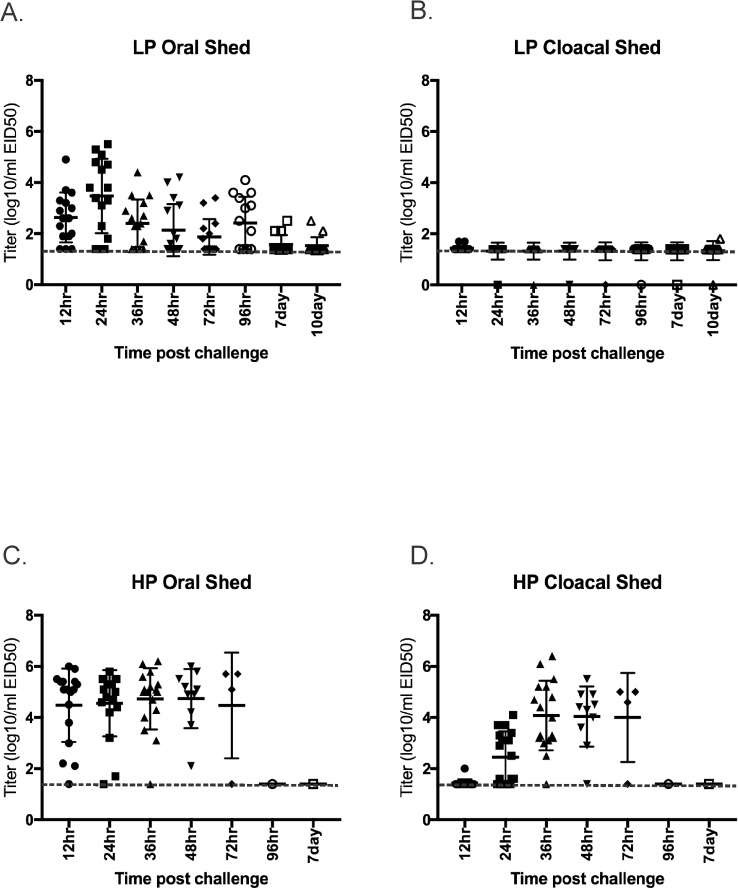
Virus shed detected by qRRT-PCR from 3 week-old chickens directly inoculated with 10^6^ 50% egg infectious doses per bird of H7N8 avian influenza viruses. A) Oropharyngeal swabs from low pathogenic avian influenza virus exposed birds (n = 17); B) Cloacal swabs from low pathogenic avian influenza virus exposed birds (n = 17); C) Oropharyngeal swabs from highly pathogenic avian influenza virus exposed birds (n = 17); D)) Oropharyngeal swabs from highly pathogenic avian influenza virus exposed birds (n = 17). Bars represent mean and standard deviation; a dotted line represents the approximate limit of detection; samples where virus was not detected are shown at the limit of detection.

No chickens were infected in the lowest dose group and 60% and 100% were infected in the medium and high dose groups respectively; the 50% bird infectious dose (BID_50_) was 10^4.0^ EID_50_ (Table 1). No contact chickens were infected in any of the dose groups based on serology and lack of virus detection in swabs. Virus was not detected in OP or CL swabs from chickens inoculated with the lowest dose, or in CL swabs from chickens inoculated with the medium dose. At the high dose, 76% of chickens shed virus by the OP route starting at 12hr PI (Fig 1A). Virus was detected in OP swabs from all but 1 of 17 chickens at multiple time points, but virus was only detected in CL swabs from 3 of 17 chickens and only at one time point each (Fig 1B).

The LPAIV isolate did not cause clinical disease in infected chickens and no gross lesions were observed in the two chickens necropsied at 2d PI from the high dose group. However, mild lymphocytic rhinitis and tracheitis were observed in both chickens and was associated with infrequent AIV antigen staining in the nasal turbinates and tracheal epithelial cells and infiltrating macrophage visualized by IHC.

### Infectivity, transmission and pathogenicity of the H7N8 LPAIV in turkeys

**Fig 2:**
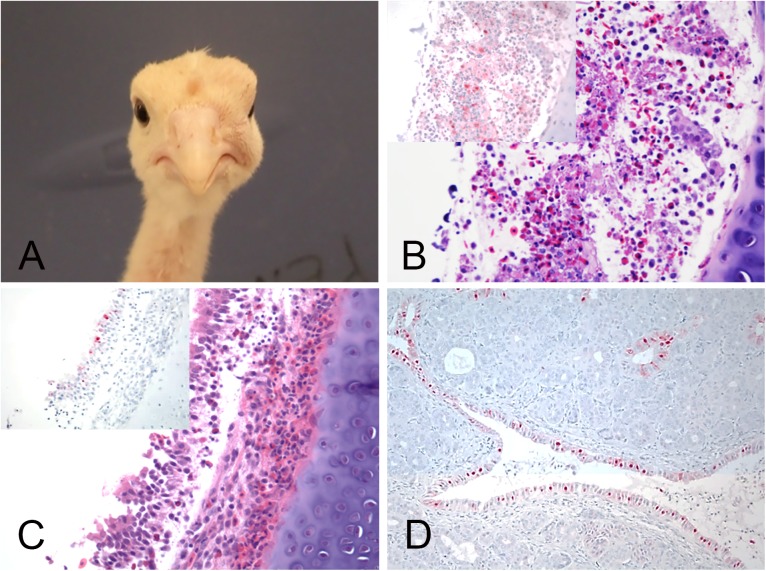
Clinical presentation, histopathology and immunohistochemical staining for AIV antigen in tissues of turkeys infected with the H7N8 LPAIV, 2days post infection. All photomicrographs, magnification 40X; hemptoxylin and eosin staining (B-C); immunohostchemistry (insets in A-B, and D), virus staining in red. A. Turkey with unilateral periorbital swelling. B. Nasal epithelium. Severe necrotizing rhinitis. Inset: Nasal epithelium, same area. Viral antigen in epithelial cells and debris, and infiltrating mononuclear cells. C. Trachea. Epithelium necrosis. Inset: Trachea, same area. Viral antigen staining in epithelial cells. D. Salivary glands. Viral antigen in the epithelial cells.

Turkeys inoculated with the LPAIV isolate were not infected in the lowest dose group, however 100% were infected in the medium and high dose groups, resulting in a BID_50_ of 10^3^ EID_50_ (Table 1). Similarly, 100% of the contacts in the medium and high dose groups were infected. Clinical signs consisting of respiratory disease (e.g. rales and “snicking”), mild lethargy, and unilateral infraorbital swelling (Fig 2) were observed in both the medium and high dose groups. No mortality and no other gross lesions, apart from that observed in sinuses, were observed in the two turkeys that were euthanized and necropsied. Microscopic lesions present in tissues from LPAIV infected turkeys included moderate to severe lymphoplasmacytic rhinitis, sinusitis and tracheitis, and mild interstitial pneumonia, and were associated with viral antigen staining in epithelial cells, vascular endothelial cells, and infiltrating mononuclear cells in nasal turbinates, trachea and salivary glands (Fig 2).

**Fig 3:**
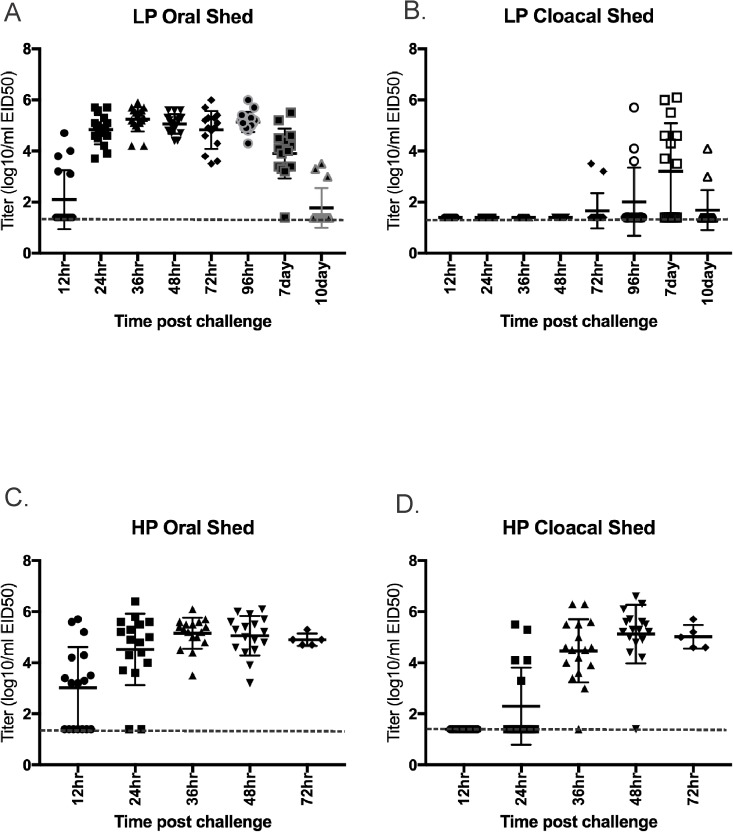
Virus shed detected by qRRT-PCR from 3 week-old turkeys directly inoculated with 10^6^ 50% egg infectious doses per bird of H7N8 avian influenza viruses. A) Oropharyngeal swabs from low pathogenic avian influenza virus exposed birds (n = 17); B) Cloacal swabs from low pathogenic avian influenza virus exposed birds (n = 17); C) Oropharyngeal swabs from highly pathogenic avian influenza virus exposed birds (n = 17); D)) Oropharyngeal swabs from highly pathogenic avian influenza virus exposed birds (n = 17). Bars represent mean and standard deviation; a dotted line represents the approximate limit of detection; samples where virus was not detected are shown at the limit of detection.

Turkeys infected with the high dose shed virus predominantly by the oral route (100%). Virus was detected at all swab sample collection times from the high dose group (Fig 3) and from the medium dose group (not shown). Although at the peak of CL virus shed the titers were similar to those detected in OP swabs, virus was not detected until 72hr PI and not from all turkeys (Fig 3).

### Infectivity, transmission and pathogenicity of the H7N8 LPAIV in mallards

Mallards inoculated with the LPAIV isolate were not infected in the lowest dose group, however 60% and 100% were infected in the medium and high dose groups, respectively, therefore the BID_50_ was 10^3.6^ EID_50_ (Table 1). Only the contacts from the high dose group were infected. No mortality and no clinical signs were observed in any of the groups. No significant differences in body weight or body temperature were observed between inoculated groups and sham inoculated mallards. No gross lesions were observed in the three mallards that were euthanized and necropsied. Microscopic lesions included moderate lymphoplasmacytic rhinitis, sinusitis and tracheitis, which was associated with viral antigen staining in the epithelial cells and infiltrating mononuclear cells of the nasal turbinates, trachea and primary bronchi.

**Fig 4:**
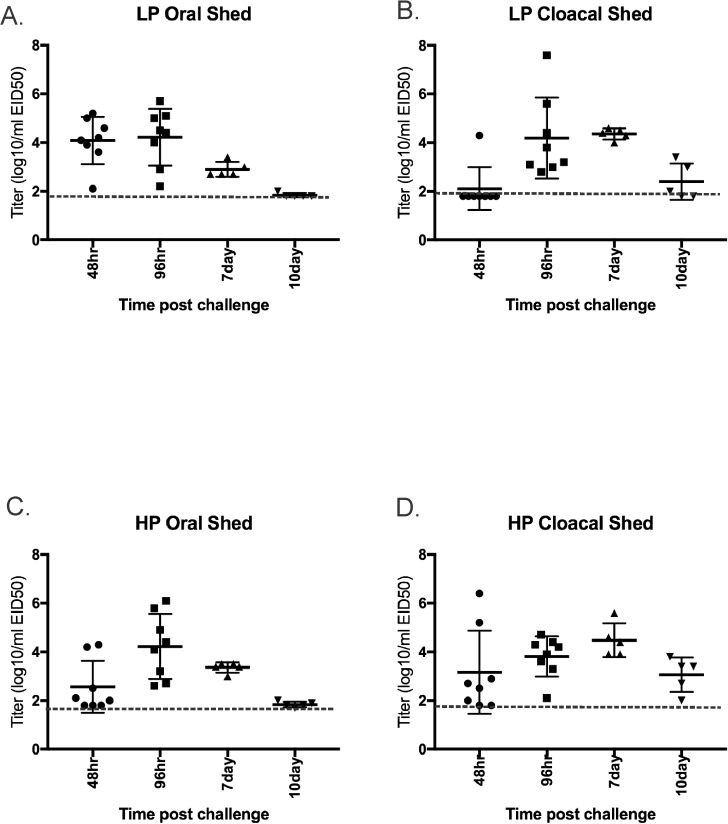
Virus shed detected by qRRT-PCR from 2 week-old mallards directly inoculated with 10^6^ 50% egg infectious doses per bird of H7N8 avian influenza viruses. A) Oropharyngeal swabs from low pathogenic avian influenza virus exposed birds (n = 8); B) Cloacal swabs from low pathogenic avian influenza virus exposed birds (n = 8); C) Oropharyngeal swabs from highly pathogenic avian influenza virus exposed birds (n = 8); D)) Oropharyngeal swabs from highly pathogenic avian influenza virus exposed birds (n = 8). Bars represent mean and standard deviation; a dotted line represents the approximate limit of detection; samples where virus was not detected are shown at the limit of detection.

Mallards infected with the high dose shed similar titer of virus by both the OP and CL route and virus was detected as late as 10d PI in OP and CL swabs (Fig 4).

### Infectivity, transmission and pathogenicity of the H7N8 HPAIV in chickens

**Fig 5:**
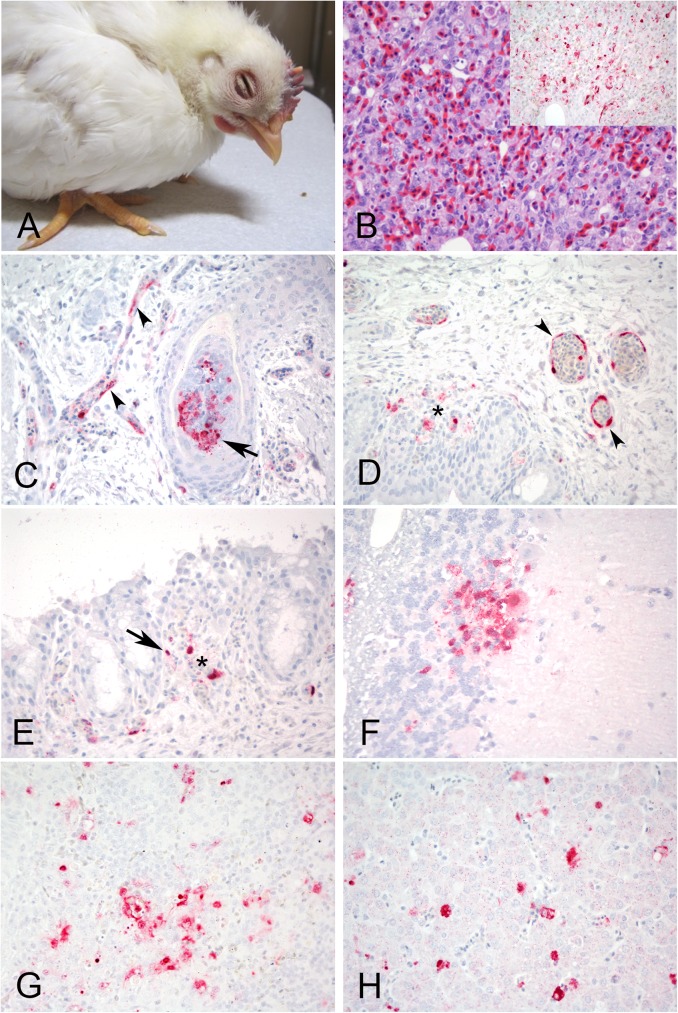
Clinical presentation, histopathology and immunohistochemical staining for AIV antigen in tissues of chickens infected with the H7N8 HPAIV, 2days post infection. All photomicrographs, magnification 40X; hematoxylin and eosin staining (B); immunohistochemistry (inset in A, and C-H), virus staining in red. A. Chicken with lethargy, swollen head, and cyanotic combs. B. Lung. Severe interstitial pneumonia. Inset: Lung, same area. Viral antigen staining in epithelium of air capillaries, mononuclear cells, and necrotic debris. C. Comb. Viral antigen staining in vascular endothelial cells (arrow head) and feather follicle epithelium (arrow). D-E. Nasal epithelium. Demonstration of viral antigen in vascular endothelial cells (arrow head), epithelial cells (arrow), and infiltrating mononuclear cells (asterisk). F. Cerebellum. Viral antigen staining in neurons and glial cells. G. Spleen. Viral antigen staining in mononuclear cells. H. Liver. Viral staining in Kupffer cells, hepatocytes, and macrophages.

Forty percent of the chickens were infected in the lowest dose group with the HP isolate, but 100% were infected in the medium and high dose groups, resulting in a BID_50_ of 10^3.2^ EID_50_ (Table 1). Similar to the LP isolate, no contact chickens were infected. Most birds died without showing clinical signs (disease was peracute). Some chickens presented with ruffled feathers, lethargy, anorexia, prostration, swollen heads, and cyanotic combs (Fig 5). Green diarrhea was also observed. Gross lesions included: empty intestines, mild to moderate splenomegaly with parenchymal mottling, and enlarged kidneys. Petechial hemorrhages were observed in the eyelid of one chicken.

Histological lesions and viral antigen staining in tissues are shown in Table 2 and Fig 5. Viral antigen staining was present in multiple tissues of infected chickens indicating systemic infection. Similar types and severity of histological lesions were observed in both birds examined: moderate to severe, multifocal necrosis was present in the parenchymal cells of many tissues but especially in lung, heart, spleen, and adrenal gland, and in some cases accompanied with mild to severe inflammation.

**Table 2:** Microscopic lesions and viral antigen distribution in tissues from chickens and turkeys intra-choanally inoculated with H7N8 HPAIV and sampled at 2d PI.

	Chickens^a^				Turkeys^a^			
Tissue	Histo score^b^	Lesions	IHC score^c^	Cell types expressing virus antigen	Histo score^b^	Lesions	IHC score^c^	Cell types expressing virus antigen
Nasal	++/++	Epithelial cell necrosis and desquamation, rhinitis, sinusitis, mononuclear cell infiltrate	++/++	Vascular endothelial cells, Nasal epithelial cells, nasal gland epithelium, mononuclear cells	+++/+++	Epithelial cell necrosis and desquamation, rhinitis, sinusitis, lymphocytic infiltrate	+++/+++	Epithelial cells, nasal gland epithelium, mononuclear cells
Trachea	++/+	Focal necrosis with mild lymphoplasmacytic inflammatory infiltrate	++/+	Pseudostratified epithelial cells, vascular endothelial cells	+/+	Focal necrosis with mild lymphoplasmacytic inflammatory infiltrate	+/+	Epithelial cells, cellular debris
Lung	+++/+++	Interstitial pneumonia with edema, congestion, necrosis, monocytic infiltrate	+++/+++	Epithelium of air capillaries, mononuclear cells, necrotic debris	++/++	Interstitial pneumonia. Bronchitis. Edema, congestion, necrosis, monocytic infiltrate	++/++	Epithelium of air capillaries, mononuclear cells, necrotic debris
Comb	++/++	Edema, hemorrhages, necrosis	++/++	Vascular endothelial cells, mononuclear cells, necrotic debris, feather follicle epithelium	+/+	Edema	+/+	Mononuclear cells, necrotic debris
Eye lid	+/+	Subcutaneous edema	+/+	Vascular endothelial cells, mononuclear cells	-/-	NA^d^	-/-	NA
Heart	+/+	Focal necrosis of myocytes	++/+++	Myocytes	++/++	Multifocal necrosis of myocytes	+++/+++	Myocytes
Brain	+/+	Neuronal necrosis, gliosis. Chromatolysis of Purkinge cell layer	++/++	Neurons, Purkinje cells, ependymal cells, glial cells, endothelial cells	+++/+++	Neuronal necrosis, gliosis. Encephalomalacia, chromatolysis of Purkinge cell layer, lymphoplasmacytic infiltrate	+++/+++	Neurons, Purkinje cells, ependymal cells, glial cells, endothelial cells
Proventriculus	-/-	NA	-/-	NA	++/-	Inflammatory infiltration in submucosa	++/-	Proventricular glandular epithelium, Mononuclear cells in submucosa
Intestine	+/+	Lymphohistiocytic infiltration in submucosa.	+/+	Mononuclear cells in lymphoid associated tissue	++/+	Lymphohistiocytic infiltration in submucosa. Epithelial necrosis	++/+	Mononuclear cells in lymphoid associated tissue
Pancreas	+/+	Mild degeneration of individual pancreatic acinar cells	+/+	Pancreatic acinar cells	++/++	Moderate degeneration of pancreatic acinar cells	++/++	Pancreatic acinar cells
Liver	-/++	Focal necrosis with lymphoplasmacytic inflammatory infiltrate	-/++	Kupffer cells, hepatocytes, endothelial cells, macrophages	+/+	Focal necrosis with lymphoplasmacytic inflammatory infiltrate	+/+	Kupffer cells, hepatocytes, endothelial cells, macrophages
Spleen	+++/+++	Multifocal areas of necrosis, hemorrhages, lymphoid depletion, hyperplasia of macrophage-phagocytic cells	+++/+++	Mononuclear cells	+++/+++	Multifocal areas of necrosis, hemorrhages, lymphoid depletion, hyperplasia of macrophage-phagocytic cells	+++/+++	Mononuclear cells, necrotic debris
Thymus	++/++	Focal necrosis, mild lymphocyte depletion, apoptotic lymphocytes	++/++	Mononuclear cells	+/+	Focal necrosis, mild lymphocyte depletion, apoptotic lymphocytes	+/+	Mononuclear cells
Cloacal bursa	+/+	Lymphocyte necrosis and apoptosis. Lymphocyte depletion, phagocytic hyperplasia	++/+	Mononuclear cells	++/++	Lymphocyte necrosis and apoptosis. Lymphocyte depletion, phagocytic hyperplasia	++/++	Mononuclear cells
Kidney	-/+	Focal necrosis of tubular epithelium with lymphoplasmacytic inflammation	+/+	Tubular epithelial and glomerular cells	++/++	Multifocal necrosis of tubular epithelium with lymphoplasmacytic inflammation	+++/++	Tubular epithelial and glomerular cells
Ovaries	-/-	NA	+/+	Tegument/interstitial tissue	+	lymphoplasmacytic infiltrate	++/+	Tegument/interstitial tissue, ova epithelium
Adrenal gland	+++/+++	Multifocal areas of necrosis with mononuclear inflammatory infiltrate	+++/+++	Corticotrophic and corticotropic cells	+++/+++	Multifocal areas of necrosis with mononuclear inflammatory infiltrate	++/++	Corticotrophic cells
Skeletal Muscle	-/-	NA	+/+	Myocytes	-/-	NA	+/+	Myocytes

a. Tissues collected from 2 birds per species (bird 1/bird2).

b. Histopathology score: histologic lesions:— = no lesions; + = mild; ++ = moderate; +++ = severe.

c. IHC = immunohistochemical staining:— = no antigen staining; + = infrequent; ++ = common; +++ = widespread.

d. NA = Not applicable

Viral antigen was detected by IHC in most tissues, with comparable patterns of tissue distribution in both chickens from which tissues were examined (Table 2, Fig 5). The presence of viral antigen was often associated with histologic lesions, although antigen was also present in areas without observable microscopic lesions. Viral antigen was frequently detected in vascular endothelial cells in the nasal cavity, trachea, eyelid and comb, while in all other tissues viral antigen was only detected in a few, individual vascular endothelial cells. Staining for viral antigen was present in areas of necrosis and in infiltrating mononuclear cells in many tissues including lymphoid tissues, lung, brain, liver, adrenal gland, and spleen, as well as in parenchymal cells of some organs, including cardiac myocytes, Kupffer cells, hepatocytes, microglial cells and neurons, epithelium of air capillaries in the lung, kidney tubular epithelial and glomerular cells, and feather follicle epithelial cells.

### Infectivity, transmission and pathogenicity of the H7N8 HPAIV in turkeys

**Fig 6:**
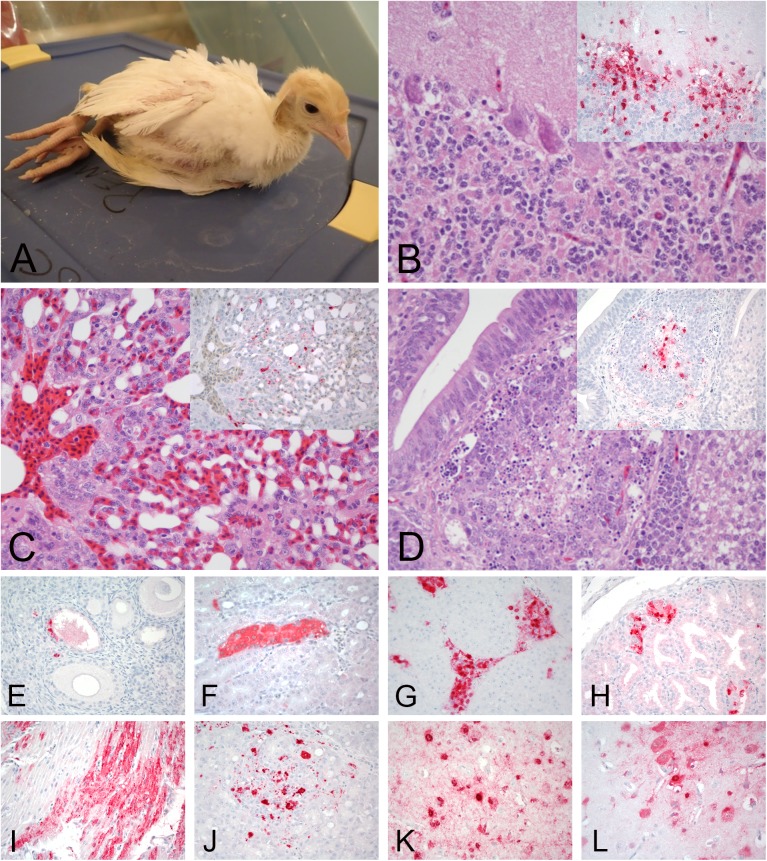
Clinical presentation, histopathology and immunohistochemical staining for avian influenza virus antigen in tissues of turkeys infected with the H7N8 HPAIV, 2days post infection. All photomicrographs, magnification 40X; hematoxylin and eosin staining (B-D); immunohostchemistry (insets in B-D, and E-L), virus staining in red. A. Turkey with neurological signs. B. Cerebellum. Severe multifocal neuronal necrosis. Insert: Cerebellum, same area. Viral antigen staining in neurons, Purkinje cells, and glial cells. C. Lung. Moderate lymphoplasmacytic interstitial pneumonia. Inset: Lung, same area. Viral antigen staining in epithelium of air capillaries and mononuclear cells. D. Cloacal bursa. Lymphoid depletion with necrotic/apoptotic lymphocytes. Inset: Cloacal bursa, same area. Viral staining in macrophages in the medulla. E. Ovary. Viral staining in tegument/interstitial tissue and ova epithelium. F. Kidney. Viral staining in tubular epithelial cells. G. Adrenal gland. Viral staining in corticotrophic cells. H. Proventriculus. Viral antigen staining in epithelium of the proventricular gland. I. Heart. Viral staining in myocytes. J. Pancreas. Viral antigen staining in mononuclear cells. K and L. Cerebrum. Viral staining in neurons and glial cells.

All turkeys in every dose group were infected, resulting in a BID_50_ of <10^2^ EID_50_. Additionally, all contact turkeys were infected regardless of dose group. Virus was first detected orally at 12hr PI and cloacally at 24hr PI and continued until death (Fig 3). Clinical signs were observed in all dose groups and consisted of neurological signs (tremors, torticollis, ataxia, opisthotonus, drooping wings) (Fig 6), green diarrhea and lethargy, which became more severe prior to death. All turkeys that were inoculated with the HPAIV isolate, or that were contact exposed, died (or were euthanized for humane reasons). There was a trend to later MDT’s in the groups that were inoculated or exposed to lower doses (Table 1). Gross lesions in dead and euthanized turkeys were primarily associated with anorexia and dehydration (e.g. empty intestines and swollen kidneys).

Histological lesions and virus staining in tissues were similar to those observed in chickens, but lesions and virus staining in the nasal cavity, brain, heart, proventriculus, pancreas, kidney, and oviduct were more severe and widespread in the turkeys. However, lesions in the lung were less severe in the turkey than in the chickens (Table 2 and Fig 6).

### Infectivity, transmission and pathogenicity of the H7N8 HPAIV in mallards

**Fig 7:**
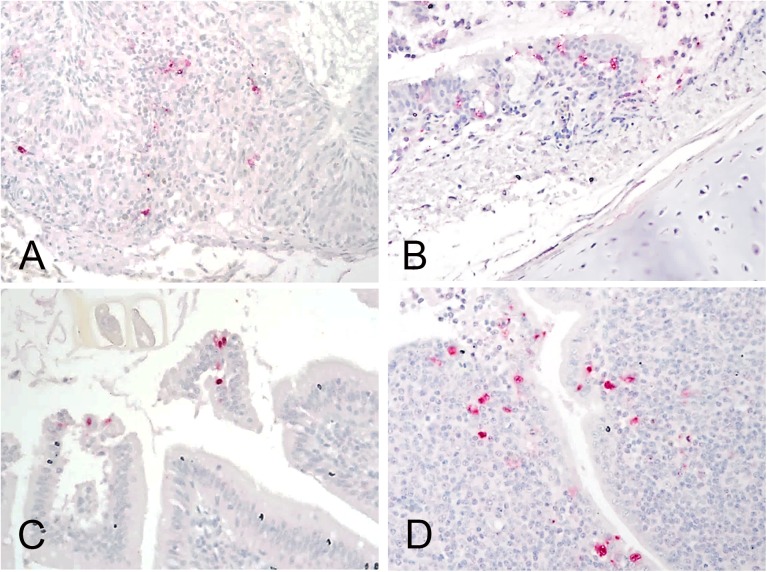
Immunohistochemical staining for AIV antigen in tissues of mallards infected with the H7N8 HPAIV, 2days post infection. All photomicrographs, magnification 40X; virus staining in red. A. Nasal epithelium. Demonstration of viral antigen in the epithelial cells and infiltrating mononuclear cells. B. Trachea. Viral antigen staining in epithelial cells. C. Intestine. Viral antigen in the epithelial cells in villi. D. Cecal tonsils. Viral antigen in the epithelial cells and mononuclear cells.

Mallards inoculated with HPAIV were infected even in the lowest dose group (40% infected); and 80% and 100% were infected in the medium and high dose groups, respectively, therefore the BID_50_ was 10^2.5^ EID_50_ (Table 1). One contact mallard from the low dose group, one from the medium dose group, and all three from the high dose group became infected. No mortality or clinical signs were observed in any of the mallards. No significant differences in temperature or body weights were observed between inoculated groups or the sham inoculated mallards (data not shown). No gross lesions were observed in the three mallards that were euthanized and necropsied. Microscopic lesions included moderate lymphoplasmacytic rhinitis, sinusitis and tracheitis and were associated with viral antigen staining in the epithelial cells and infiltrating mononuclear cells of the nasal turbinates and trachea (Fig 7). Viral antigen was also found in epithelial cells of the air sac, intestine and bursa of Fabricius, and in isolated, single cells in lung, hert and spleen.

Similar to the LP isolate, mallards shed virus by both the OP and CL route with prolonged shedding by the CL route (Fig 4).

### LPAIV versus HPAIV virus shed titers

Oral and CL shed titers between LPAIV and HPAIV infected birds were compared among all 3 species in the high dose groups. In chickens, HPAIV titers were significantly higher than LPAIV orally from 12 to 48hrs PI and cloacally from 24 to 48hrs PI (S1 Fig). In turkeys, LPAIV and HPAIV shed titers were statistically not different at most time points, and were only significantly higher with HPAIV cloacally 24-48hrs PI (S2 Fig). Too few chickens and turkeys exposed to HPAIV were alive at 72hrs and later for accurate statistical analysis. In mallards, LPAIV shed titers were significantly higher at 48hrs orally, and HPAIV titers were higher at 48hr cloacally and at 7d orally (S3 Fig).

With both chickens and turkeys the proportion of birds shedding virus cloacally was significantly higher in the HPAIV exposed birds at 24, 36, 48 and 72 hr PI and statistically equal at 12hr PI (too few HPAIV exposed birds survived for comparison at later time points). With oral shed, the only time when the proportion of birds shedding virus was significantly different between HPAIV and LPAIV was at 48hr PI when more HPAIV inoculated chickens were shedding virus. In mallards there were no differences between the AIV phenotypes in the proportion shedding virus.

### Virus shed titers differences by species

The shed titers within a pathotype were compared among chickens, turkeys and mallards for both LPAIV and HPAIV. With LPAIV, chickens shed significantly more virus orally than turkeys at 12hr PI, but turkeys shed significantly higher titers at every other time point through 7d PI (S4 Fig). Turkeys also shed higher virus titers than mallards at 48hr PI orally with both LPAIV and HPAIV (S4 and S5 Figs). However, mallards shed higher titers cloacally with the LPAIV isolate at 48hr PI. At later time points turkeys shed significantly higher titers orally (96hr and 7d) and mallards shed higher titers cloacally (96hr and 10d PI). Regardless of statistical significance there was a general trend for turkeys and mallards to shed similar titers, which were often greater than titers from chickens.

The proportion of birds shedding had a similar pattern to shed titers. With LPAIV, significantly more chickens were shedding at 12hr PI, and significantly more turkeys were shedding virus at 48hr, 72hr, 96hr and 7d PI. More mallards than chickens were shedding LPAIV orally (48hr, 7d) and cloacally (96hr, 7d and 10d PI). By the CL route the only difference was at 7d PI when significantly more turkeys were shedding than chickens. With HPAIV, significantly more chickens were shedding at 12hr by the oral route and at 24hr PI by the CL route.At all other times the highest proportion shedding virus were either the turkeys or mallards. The overall trend was that mallards and turkeys were similar with more birds shedding virus than chickens.

### Virus replication in tissues

Virus replication was examined by qRRT-PCR at 2-3d PI in brain, heart, lung, spleen, and muscle tissue from chickens, turkeys and mallards following intranasal inoculation with the H7N8 LPAIV and HPAIV. Tissues from one turkey were examined at 6d PI as well. High viral titers were detected in all tissues from chickens and turkeys inoculated with the HPAIV, indicating extensive systemic viral replication (Table 3). The LPAIV was only detected in the spleen of one chicken but was detected in all the turkey tissues examined, but viral titers were 3–4 log_10_ lower than the observed with the HPAI virus. In mallards, LPAIV was only detected in the brain and muscle of one mallard of three examined. Moderate to high HPAIV titers were detected in all tissues of all three mallards examined (Table 3), but titers were lower than those found in tissues from chickens and turkeys.

**Table 3:** Virus titers in log_10_ 50% egg infectious doses per gram (EID_50_/g) in tissues from chickens, turkeys and mallards inoculated with H7N8 LPAIV or H7N8 HPAIV. The threshold of detection in tissue was 10^1.5^ EID_50_/g.

Species	Bird ID	Pathotype	Day Post challenge	Titer (log_10_ EID_50_/g)				
				Lung	Spleen	Heart	Brain	Muscle
Chicken	1	LP	2	-^a^	-	-	-	-
Chicken	2	LP	2	-	1.8	-	-	-
Chicken	1	HP	2	7.6	6.5	6.8	7.5	8.0
Chicken	2	HP	2	6.6	7.9	7.1	7.2	7.4
Turkey	1	LP	2	4.8	3.1	4.6	3.7	4.8
Turkey	2	LP	2	3.8	4.1	4.7	5.4	4.8
Turkey	1	HP	2	8.8	8.4	9.3	>9.6	9.1
Turkey	2	HP	2	8.9	8.1	9.2	9.5	9.0
Turkey	3	HP	6	7.8	8.4	9.1	9.6	7.2
Mallard	1	LP	3	-	-	-	-	-
Mallard	2	LP	3	-	-	-	5.1	2.5
Mallard	3	LP	3	-	-	-	-	-
Mallard	1	HP	3	4.5	3.6	3.6	3.7	3.0
Mallard	2	HP	3	4.8	4.4	6.2	4.9	6.0
Mallard	3	HP	3	6.9	4.7	6.8	NA	6.6

a.– = negative. NA = not available

## Discussion

In January 2016, H7N8 HPAIV and LPAIV were isolated from commercial turkeys in Indiana, USA. The pathogenesis of both pathotypes were evaluated in chickens, turkeys and mallards and revealed species specific differences in the outcome of infection. The MDT for the HPAIV was the same for chickens and turkeys at the highest dose, but was 30% longer in turkeys than chickens at the medium dose. In contrast there was no mortality in the mallards. This finding is consistent with the differences in shed patterns among the two species; chickens shed high titers of virus early after inoculation, but after 24hr levels were consistently higher from turkeys. Similarly, a higher proportion of turkeys and mallards shed detectable levels of LPAIV versus chickens at numerous time points. In addition to transmissibility, virus shed patterns have a practical impact on virus detection, as higher shed titers and higher number of birds shedding improve the likelihood of detecting the virus in affected flocks.

Clinical signs also varied among the three species. Neither chickens nor mallards exhibited clinical signs when infected with LPAIV, but turkeys presented with respiratory disease. Although the severity of disease was mild-to-moderate among the turkeys in our study, infection of turkeys in the field could be exacerbated by secondary infection or environmental stress [14–16]. Previous reports of LPAIV in turkeys and chickens demonstrate that the severity of disease can be highly variable in the field, but is typically mild if uncomplicated [15, 17–21]. Similarly the absence of clinical disease is common with LPAIV infection in chickens or ducks [7]. More severe disease with LPAIV infection in turkeys versus Pekin ducks and chickens in side-by-side comparisons has been reported [22]. Some have reported that disease severity is similar, but in that case chickens shed higher titers and turkeys were susceptible to infection with more LPAIV isolates at a higher dose (10^6^ EID_50_ per bird) [23]. A third study showed that the LPAIV’s examined transmitted better between turkeys and that the infectious doses were lower in turkeys compared to chickens and ducks [24].

Although the outcome of AIV exposure is reliant upon the specific isolate-species combination, in general and as corroborated in this study, turkeys appear to be more susceptible to AIV infection than chickens or mallards. In our study, with some exceptions (e.g. the lung and comb) broader tissue tropism and higher viral titers where observed in the turkeys when compared to chickens and mallards, as seen by both IHC and qRRT-PCR. Interestingly, LPAIV was detected by qRRT-PCR in several internal organs in infected turkeys as well. The specific cells in which the virus was replicating within these tissues could not be identified by IHC because of the insensitivity of this assay (titers of ≥10^6^ EID_50_/g are typically required to be able to visualize the virus by this IHC method in tissues).

The HPAIV isolate did not cause clinical disease in mallards, which is expected because most HPAIV strains do not cause disease in mallards or their domestic counterpart, Pekin ducks (*Anas platyrhynchos domesticus*), with the exception of some strains of the A/goose/Guangdong/1/1996 lineage of H5 HPAIVs [25, 26]. Severe disease with HPAIV infection was observed with chickens and turkeys, as expected. However, the presentation of disease varied between these two species. Disease in chickens was similar to what has been reported before with other HPAIV’s; i.e. early death with minimal clinical signs (peracute disease), infraorbital swelling, ruffled feathers, cyanotic combs, hemorrhagic lesions, and severe lethargy [7]. In turkeys, infection with HPAIV presented differently; neurological signs were observed in most birds and no hemorrhagic lesions were present. In other field and experimental reports, neurological signs are commonly mentioned for turkeys and, unlike chickens, hemorrhagic lesions on the shanks are not reported [16, 27–29]. In both chickens and turkeys the most common gross lesions were non-specific and were likely due to the birds not eating or drinking (i.e. empty intestines and swollen kidneys).

The infectivity and intraspecies transmission of the viruses varied among the three species. Chickens were the most resistant to infection with either the LP or the HPAIV, and turkeys were the most susceptible, based on BID_50_ and spread to contact exposed hatch-mates. The relatively high susceptibility of turkeys to LPAIV compared to chickens and ducks has been reported in previous studies, although there are exceptions and differences in the specific BID_50_ among isolates [24, 30–34]. We have not found a report where the BID_50_ was compared within species between LP and HPAIV, but one report did show that the A/Pennsylvania/1370/1983 H5N2 HPAIV did transmit better among chickens than the LP phenotype (A/chicken/Pennsylvania/21525/1983 H5N2 LPAIV) [35]. Here, the BID_50_ was at least 10 times lower for HPAIV than LPAIV for all three species. Increased shed titers and/or the proportion of birds shedding virus, which are consequences of increased efficiency of virus replication, could contribute to better transmission in the field. Transmission was not efficient among chickens in our study, which is likely an artifact of our housing (isolators with high rates of airflow and grate floors) and has been seen previously [32].

In contrast to the H5N2 HPAIVs that caused a much more widespread outbreak in turkeys and layer chickens in the Midwest US in 2015, when over 200 farms were infected [36], the MDT in turkeys with the H7N8 HPAIV used in this study was three days shorter [37]. A shorter MDT may aid control because the disease is recognized earlier and there is less time for the birds to shed virus into the environment. However, both the LP and HP phenotypes of this H7N8 strain had lower mean bird infectious doses and were more transmissible among turkeys than the H5N2 isolates [37]. In chickens, the H7N8 isolates also had lower mean infectious doses than the 2014 index H5N2 wild bird isolate [32], but were similar to the H5N2 viruses isolated from poultry later in the outbreak [33]. This reinforces that epidemiological links are critical for farm-to-farm virus spread regardless of virus infectivity. A low infectious dose and increased virus shedding likely increase risk of infection when there is an exposure event (i.e. epidemiological link). In many cases the epidemiological link is unknown. During the outbreak in IN, positive flocks were rapidly depopulated, which was likely a major contributing factor to the short duration of the outbreak because it limited the potential for virus contact with susceptible flocks regardless of infectious dose and virus shed titers. However, the combination of lower bird infectious dose for turkeys versus chickens and epidemiological links between turkey farms were likely responsible for the outbreak being limited to turkeys, despite the presence of chickens farms within Dubois county, Indiana and, more specifically, one epidemiologically linked dangerous contact chicken farm that was not affected.

Interestingly, in mallards, the HP isolate also had a lower mean infectious dose than the LP isolate. Recent reports demonstrate that mallards are susceptible to HPAIV infection [38]; however, some poultry-adapted viruses might be less infectious for mallards [33]. Either way, the acquisition of the HP phenotype during passage of LP viruses in gallinaceous poultry, which was thought to be a marker of increased virus adaptation in poultry, does not seem to reduce the infectivity of AIV for mallards. Importantly, the fact that mallards could become infected with both the HP and the LP H7N8 AIVs, and could efficiently transmit the virus to contacts, highlights the importance of preventing contact between infected poultry and wild waterfowl during outbreaks.

## Supporting information

S1 Fig Virus shed detected by qRRT-PCR from 3 week-old chickens directly inoculated with 10^6^ 50% egg infectious doses per bird of H7N8 avian influenza viruses by time post inoculation. A) Oro-pharyngeal swabs from low pathogenic and highly pathogenic avian influenza virus exposed birds (n = 17); B) Cloacal swabs from low pathogenic and highly pathogenic avian influenza virus exposed birds (n = 17). Bars represent mean and standard deviation; a dotted line represents the approximate limit of detection; samples where virus was not detected are shown at the limit of detection; LP = low pathogenic (shown in black), HP = highly pathogenic (shown in red). The 10day time point is not shown because insufficient chickens in the HP group were alive for statistical analysis. Brackets with an asterisk denote statistical significance at a p value of ≤ 0.05 between the bracketed groups.
(PDF)

S2 Fig Virus shed detected by qRRT-PCR from 3 week-old turkeys directly inoculated with 10^6^ 50% egg infectious doses per bird of H7N8 avian influenza viruses by time post inoculation. A) Oro-pharyngeal swabs from low pathogenic and highly pathogenic avian influenza virus exposed birds (n = 17); B) Cloacal swabs from low pathogenic and highly pathogenic avian influenza virus exposed birds (n = 17). Bars represent mean and standard deviation; a dotted line represents the approximate limit of detection; samples where virus was not detected are shown at the limit of detection; LP = low pathogenic (shown in black), HP = highly pathogenic (shown in red). The 96hr, 7day and 10day time points are not shown because insufficient turkeys in the HP group were alive for statistical analysis. Brackets with an asterisk denote statistical significance at a p value of ≤ 0.05 between the bracketed groups.
(PDF)

S3 Fig Virus shed detected by qRRT-PCR from 2 week-old mallards directly inoculated with 10^6^ 50% egg infectious doses per bird of H7N8 avian influenza viruses by time post inoculation. A) Oro-pharyngeal swabs from low pathogenic and highly pathogenic avian influenza virus exposed birds (n = 8); B) Cloacal swabs from low pathogenic and highly pathogenic avian influenza virus exposed birds (n = 8). Bars represent mean and standard deviation; a dotted line represents the approximate limit of detection; samples where virus was not detected are shown at the limit of detection; LP = low pathogenic (shown in black), HP = highly pathogenic (shown in red). Brackets with an asterisk denote statistical significance at a p value of ≤ 0.05 between the bracketed groups.
(PDF)

S4 Fig Virus shed detected by qRRT-PCR from 3 week-ld chickens (shown in black), 3 week-old turkeys (shown in blue) and 2 week-old mallards (shown in red) directly inoculated with 10^6^ 50% egg infectious doses per bird of H7N8 low pathogenic avian influenza virus by time post inoculation. A) Oro-pharyngeal swabs; B) Cloacal swabs. Bars represent mean and standard deviation; a dotted line represents the approximate limit of detection; samples where virus was not detected are shown at the limit of detection. Brackets with an asterisk denote statistical significance at a p value of ≤ 0.05 between the bracketed groups.
(PDF)

S5 Fig Virus shed detected by qRRT-PCR from 3 week-old chickens (shown in black), 3 week-old turkeys (shown in blue) and 2 week-old mallards (shown in red) directly inoculated with 10^6^ 50% egg infectious doses per bird of H7N8 highly pathogenic avian influenza virus by time post inoculation. A) Oro-pharyngeal swabs; B) Cloacal swabs. Bars represent mean and standard deviation; a dotted line represents the approximate limit of detection; samples where virus was not detected are shown at the limit of detection. Columns with no data mean that there were no birds alive in the group at that time point. Brackets with an asterisk denote statistical significance at a p value of ≤ 0.05 between the bracketed groups.
(PDF)
