# Supplementary material for: The pathogenesis of H7N8 low and highly pathogenic avian influenza viruses from the United States 2016 outbreak in chickens, turkeys and mallards
Source: PLoS One. 2017 May 8;12(5):e0177265. doi: 10.1371/journal.pone.0177265 (PMC5421793; doi:10.1371/journal.pone.0177265)
Supplement: S2 Fig — A) Oro-pharyngeal swabs from low pathogenic and highly pathogenic avian influenza virus exposed birds (n = 17); B) Cloacal swabs from low pathogenic and highly pathogenic avian influenza virus exposed birds (n = 17). Bars represent mean and standard deviation; a dotted line represents the approximate limit of detection; samples where virus was not detected are shown at the limit of detection; LP = low pathogenic (shown in black), HP = highly pathogenic (shown in red). The 96hr, 7day and 10day time points are not shown because insufficient turkeys in the HP group were alive for statistical analysis. Brackets with an asterisk denote statistical significance at a p value of ≤ 0.05 between the bracketed groups. (PDF) [file pone.0177265.s002.pdf]

Supplemental figures for: Pantin-Jackwood, et al. *The pathogenesis of H7N8 low and highly pathogenic avian influenza viruses from the United States 2016 outbreak in chickens, turkeys and mallards*

A.

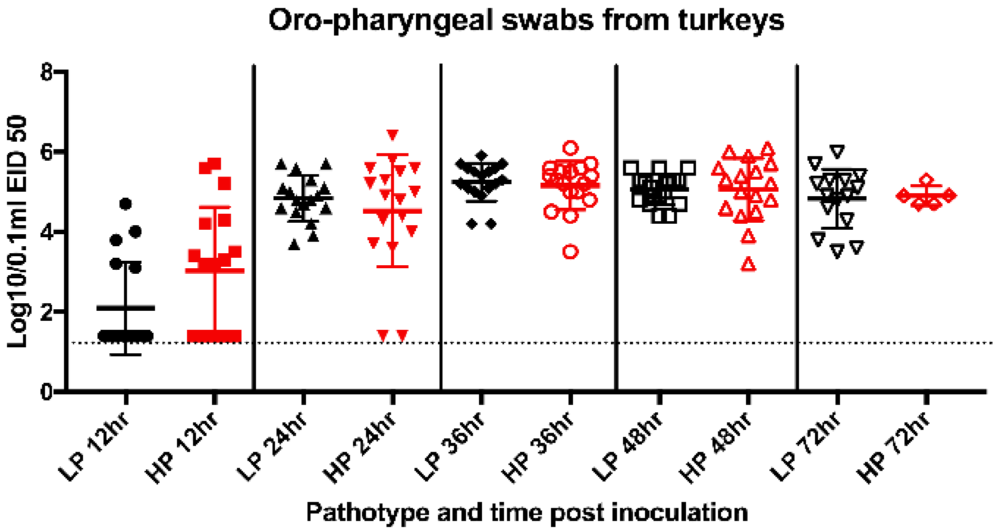

B.

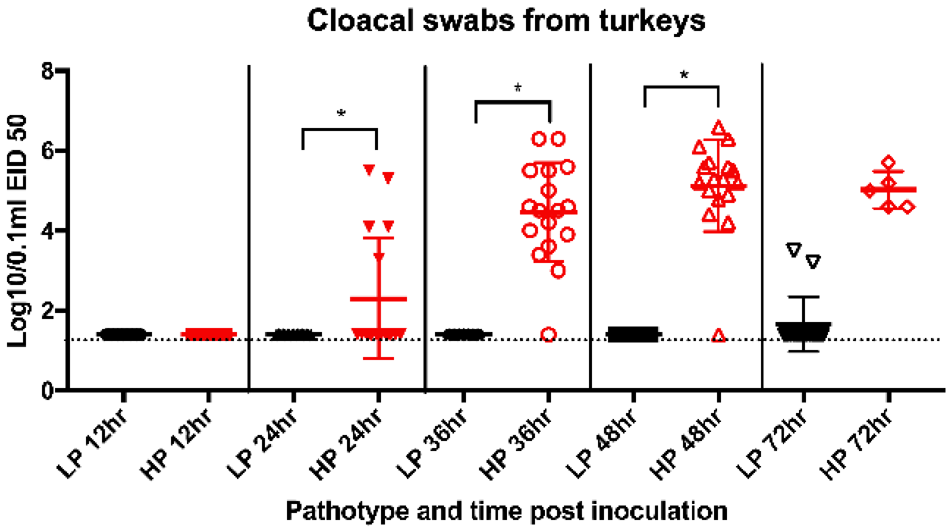

Supplemental Figure 2. Virus shed detected by qRRT-PCR from 3 week-old turkeys directly inoculated with 10^6 50% egg infectious doses per bird of H7N8 avian influenza viruses by time post inoculation: A) Oro-pharyngeal swabs from low pathogenic and highly pathogenic avian influenza virus exposed birds (n=17); B) Cloacal swabs from low pathogenic and highly pathogenic avian influenza virus exposed birds (n=17). Bars represent mean and standard deviation; a dotted line represents the approximate limit of detection; samples where virus was not detected are shown at the limit of detection; LP=low pathogenic (shown in black), HP=highly pathogenic (shown in red). The 96hr, 7day and 10day time points are not shown because insufficient turkeys in the HP group were alive for statistical analysis. Brackets with an asterisk denote statistical significance at a p value of ≤ 0.05 between the bracketed groups.
