# Supplementary material for: The pathogenesis of H7N8 low and highly pathogenic avian influenza viruses from the United States 2016 outbreak in chickens, turkeys and mallards
Source: PLoS One. 2017 May 8;12(5):e0177265. doi: 10.1371/journal.pone.0177265 (PMC5421793; doi:10.1371/journal.pone.0177265)
Supplement: S3 Fig — A) Oro-pharyngeal swabs from low pathogenic and highly pathogenic avian influenza virus exposed birds (n = 8); B) Cloacal swabs from low pathogenic and highly pathogenic avian influenza virus exposed birds (n = 8). Bars represent mean and standard deviation; a dotted line represents the approximate limit of detection; samples where virus was not detected are shown at the limit of detection; LP = low pathogenic (shown in black), HP = highly pathogenic (shown in red). Brackets with an asterisk denote statistical significance at a p value of ≤ 0.05 between the bracketed groups. (PDF) [file pone.0177265.s003.pdf]

Supplemental figures for: Pantin-Jackwood, et al. *The pathogenesis of H7N8 low and highly pathogenic avian influenza viruses from the United States 2016 outbreak in chickens, turkeys and mallards*

A.

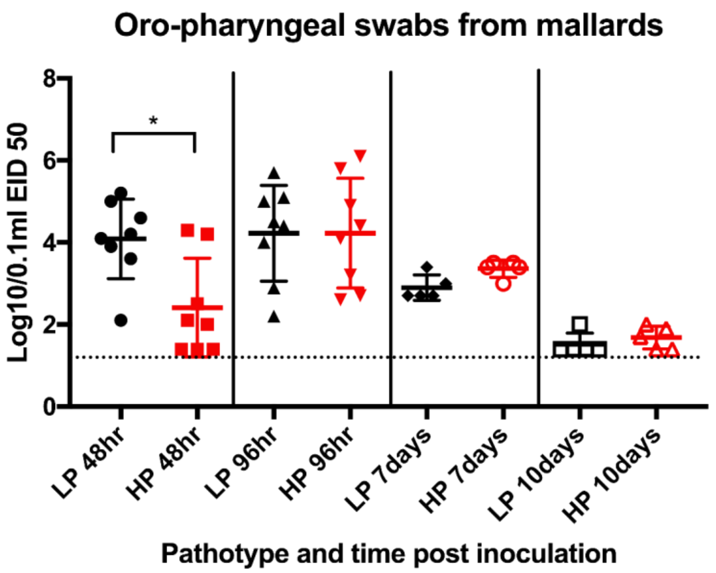

B.

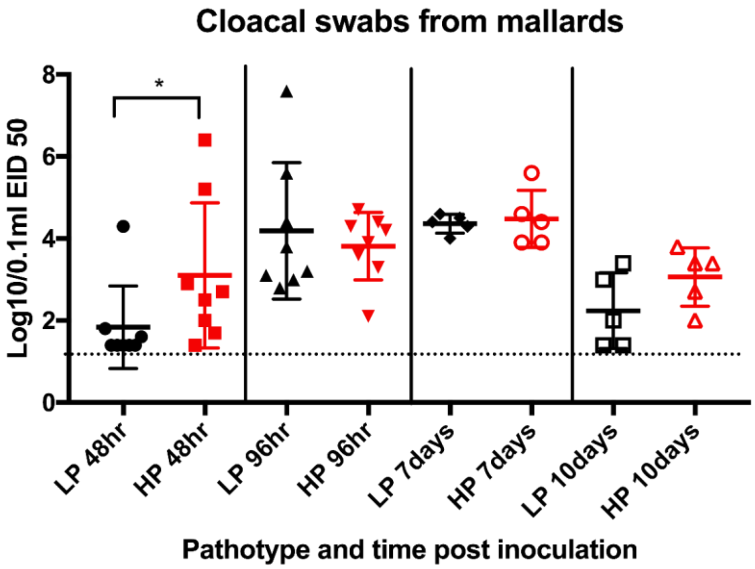

Supplemental Figure 3. Virus shed detected by qRRT-PCR from 2 week-old mallards directly inoculated with 10^6 50% egg infectious doses per bird of H7N8 avian influenza viruses by time post inoculation: A) Oro-pharyngeal swabs from low pathogenic and highly pathogenic avian influenza virus exposed birds (n=8); B) Cloacal swabs from low pathogenic and highly pathogenic avian influenza virus exposed birds (n=8). Bars represent mean and standard deviation; a dotted line represents the approximate limit of detection; samples where virus was not detected are shown at the limit of detection; LP=low pathogenic (shown in black), HP=highly pathogenic (shown in red). Brackets with an asterisk denote statistical significance at a p value of ≤ 0.05 between the bracketed groups.
