# Supplementary material for: The pathogenesis of H7N8 low and highly pathogenic avian influenza viruses from the United States 2016 outbreak in chickens, turkeys and mallards
Source: PLoS One. 2017 May 8;12(5):e0177265. doi: 10.1371/journal.pone.0177265 (PMC5421793; doi:10.1371/journal.pone.0177265)
Supplement: S4 Fig — A) Oro-pharyngeal swabs; B) Cloacal swabs. Bars represent mean and standard deviation; a dotted line represents the approximate limit of detection; samples where virus was not detected are shown at the limit of detection. Brackets with an asterisk denote statistical significance at a p value of ≤ 0.05 between the bracketed groups. (PDF) [file pone.0177265.s004.pdf]

**Supplemental figures for:** Pantin-Jackwood, et al. *The pathogenesis of H7N8 low and highly pathogenic avian influenza viruses from the United States 2016 outbreak in chickens, turkeys and mallards*

A.

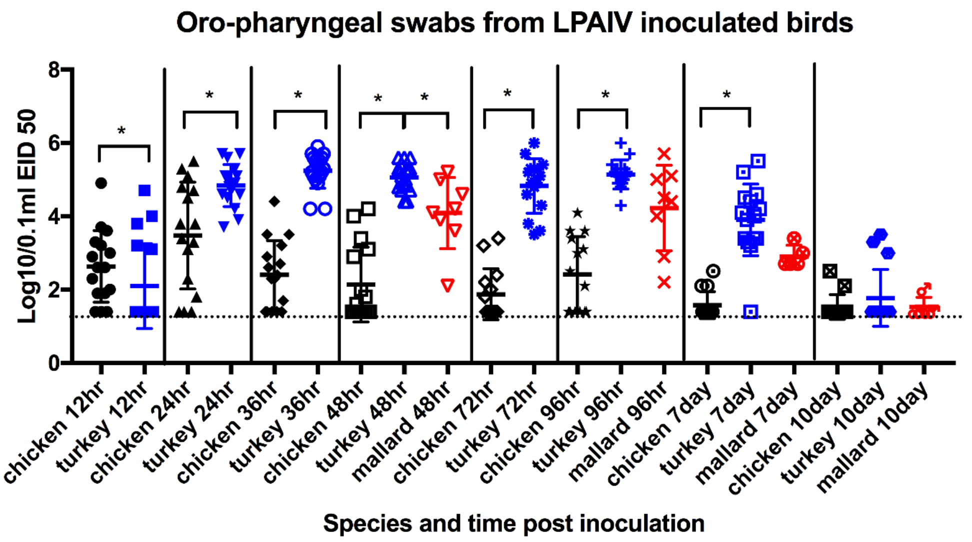


B.

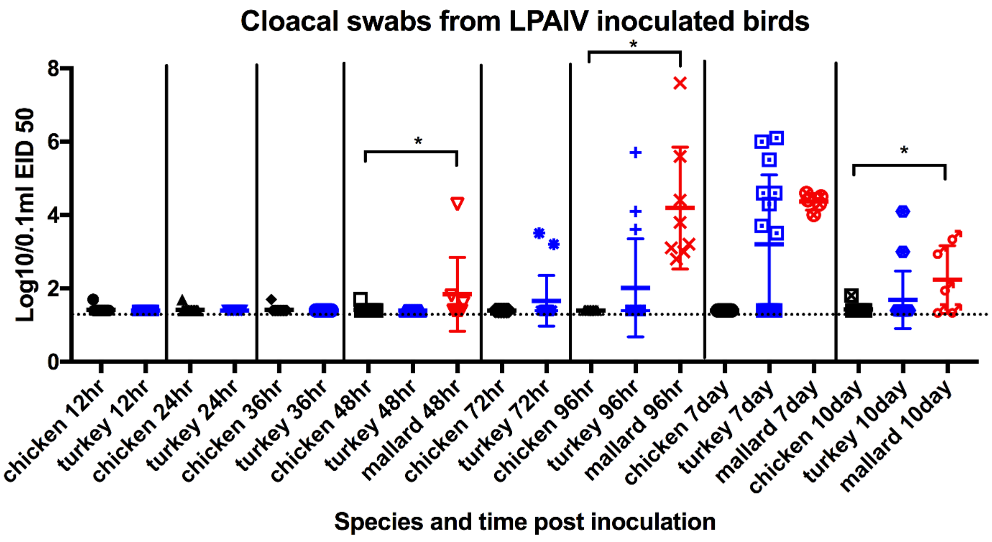


**Supplemental Figure 4.** Virus shed detected by qRRT-PCR from 3 week-ld chickens (shown in black), 3 week-old turkeys (shown in blue) and 2 week-old mallards (shown in red) directly inoculated with $10^6$ 50% egg infectious doses per bird of H7N8 low pathogenic avian influenza virus by time post inoculation: A) Oro-pharyngeal swabs; B) Cloacal swabs. Bars represent mean and standard deviation; a dotted line represents the approximate limit of detection; samples where virus was not detected are shown at the limit of detection. Brackets with an asterisk denote statistical significance at a p value of $\leq 0.05$ between the bracketed groups.
