# Supplementary material for: The pathogenesis of H7N8 low and highly pathogenic avian influenza viruses from the United States 2016 outbreak in chickens, turkeys and mallards
Source: PLoS One. 2017 May 8;12(5):e0177265. doi: 10.1371/journal.pone.0177265 (PMC5421793; doi:10.1371/journal.pone.0177265)
Supplement: S5 Fig — A) Oro-pharyngeal swabs; B) Cloacal swabs. Bars represent mean and standard deviation; a dotted line represents the approximate limit of detection; samples where virus was not detected are shown at the limit of detection. Columns with no data mean that there were no birds alive in the group at that time point. Brackets with an asterisk denote statistical significance at a p value of ≤ 0.05 between the bracketed groups. (PDF) [file pone.0177265.s005.pdf]

Supplemental figures for: Pantin-Jackwood, et al. *The pathogenesis of H7N8 low and highly pathogenic avian influenza viruses from the United States 2016 outbreak in chickens, turkeys and mallards*

A.

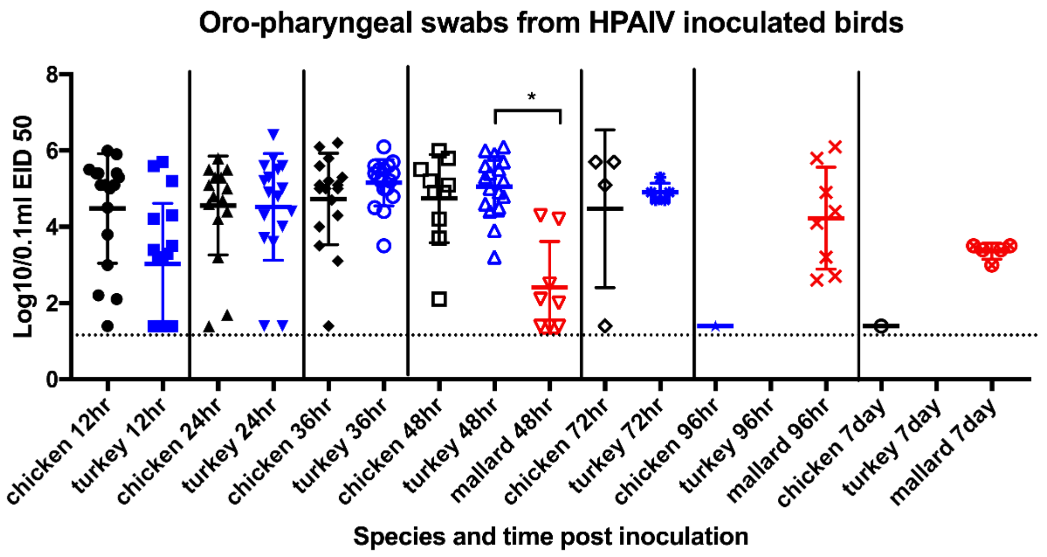

B.

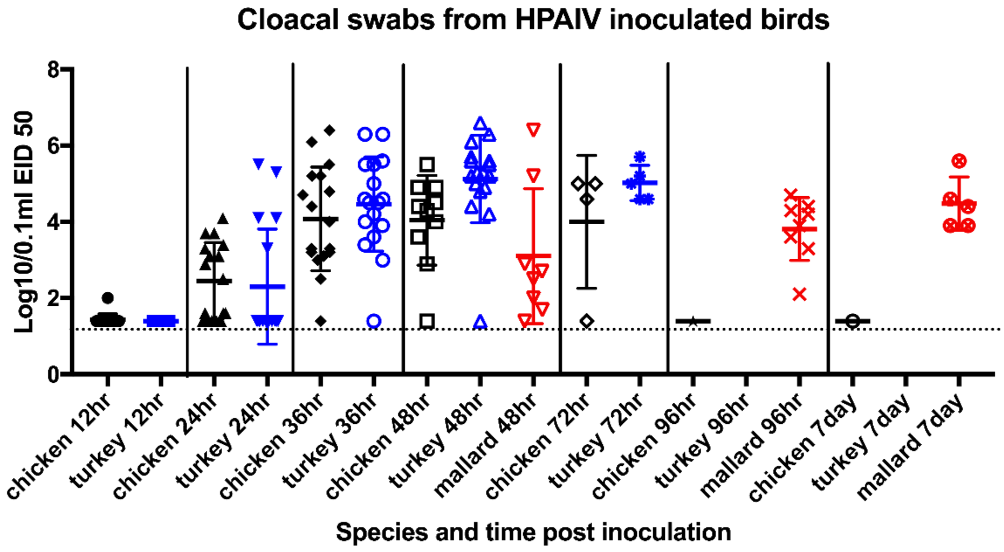

Supplemental Figure 5. Virus shed detected by qRRT-PCR from 3 week-ld chickens (shown in black), 3 week-old turkeys (shown in blue) and 2 week-old mallards (shown in red) directly inoculated with 10^6 50% egg infectious doses per bird of H7N8 highly pathogenic avian influenza virus by time post inoculation: A) Oro-pharyngeal swabs; B) Cloacal swabs. Bars represent mean and standard deviation; a dotted line represents the approximate limit of detection; samples where virus was not detected are shown at the limit of detection. Columns with no data mean that there were no birds alive in the group at that time point. Brackets with an asterisk denote statistical significance at a p value of ≤ 0.05 between the bracketed groups.
